# Supplementary material for: Study of blood glucose and insulin infusion rate in real-time in diabetic rats using an artificial pancreas system
Source: PLoS One. 2021 Jul 16;16(7):e0254718. doi: 10.1371/journal.pone.0254718 (PMC8284668; doi:10.1371/journal.pone.0254718)
Supplement: Attachment — Submitted filename: Responses to Reviewers-Final.docx [file pone.0254718.s001.docx]

**Responses to Reviewers (PONE-D-21-07395).**

We sincerely appreciate Reviewers’ excellent critiques, which helped to enhance the quality of the manuscript significantly after revision.

The revised manuscript contains significant changes in response to the critiques raised by reviewers. We added one new figure (Fig 1) and revised the whole manuscript to incorporate our responses to the comments raised by reviewers. Below are our responses to the critiques written in red, and revisions incorporated in the manuscript in blue. The page and the line numbers correspond to the marked version of the revised manuscript.

**Reviewer 1**

1. A number of antidiabetic drugs including insulin formulations have been successfully developed and launched, so far. However, the authors claimed that diurnal variation of blood glucose and insulin levels could disturb evaluation of the effects of antidiabetic agents. The authors should raise detailed case examples.

Responses: A combination of different formulations of insulin has been successfully used to treat type 1 diabetes. Recently, add on therapies with antidiabetic agents such as SGLT-2 inhibitors, GLP-1 analogs, or amylin analogs are considered to treat type 1 diabetes along with insulin. A rat APS may be used to assess the effectiveness of these agents by studying alterations of glucose-insulin regulation before, middle, and after the drug treatment or between control and treated groups. An example of evaluation of add-on therapy using a closed-loop APS is described in the Introduction section.

We have modified Introduction section by adding the example shown on p4, lines 82-85.

2. The authors conducted histological assessments and blood biochemical measurements in addition to APS development. The authors should explain about the relationship among results obtained from these experiments. In its current form, the results are not reflected in the title of this manuscript.

Responses: In addition to rat APS development, we also demonstrated its applications in studying the effects of different diets and injection of supraphysiological glucose concentrations on glucose-insulin regulation in healthy and diabetic rats. Histological assessments and blood biochemical measurements are critical to understand the physiological states of the healthy and diabetic rats used in the study to support and understand the APS data. We discussed the relationship and the importance of ex vivo studies in the Discussion section.

We have revised the manuscript incorporating our responses on p19, lines 488-491 and p19, lines 500-502 in the Discussion section.

3. The authors did not mention whether streptozotocin depleted insulin secretion or not.

Responses: Based on the immunohistochemical studies (Fig 8), stretozotocin injection caused complete or near-complete destruction of β-cells. The extent by which the remaining β-cells release insulin is unknown, but considered negligible based on the absence of β-cells in the diabetic rats.

We have revised the manuscript incorporating our responses on p19, lines 490-491.

4. The authors should conduct statistical analyses of the data shown in Table 1

Responses: We conducted paired t-test and Mann-Whitney test for statistical analysis.

We have revised the manuscript incorporating our responses in Table 1 and legend shown on p12, lines 292-294.

5. Please show units in Table 3.

Responses: The units of g for body weight and % for HbAIc had been added to Table 3.

We have revised Table 3 by adding units as described above.

6. In several figures, X-axes are not shown, and Y-axes are fuzzy.

Responses: We have remade figures to include X-axes and make Y-axes clear.

New figures were added to the revised manuscript.

7. The authors should provide a detailed description of intraperitoneal glucose injection (e.g., amount and duration of injection).

Responses: A detailed description of intraperitoneal glucose injection has been added to the Methods section.

We have revised the manuscript incorporating our responses on p7, line 158.

**Reviewer 2**

1. In the title of the manuscript, and several places in the text, you state that you have examined blood glucose and insulin levels in real time. CGM does indeed provide a representation of blood glucose values in (approximately) real-time, but I find the statement of studying insulin levels to be misleading in this context. I believe you have evaluated the insulin dosing calculated by the controller, not the insulin levels present in the rat, which would be impossible in real-time. One could argue that evaluating the insulin delivery from the controller could be an adequate estimation of the actual insulin requirement of the streptozotocin treated rats, but to state that they represent the “physiological truth”, is quite strong. That would imply the perfect controller, which we are all still working on. (But, it would have been great!!) I am sure you have made your thoughts on this and if you could include them in the discussion, it would be nice.

Responses: We agree with reviewer that we had not studied insulin ‘level’ in real-time with a rat APS. We changed the title and removed any potentially misleading statements in the manuscript.

We have revised the manuscript incorporating our responses on p1, lines 3-6.

2. What is the benefit of testing the AP on healthy rats? I understand the value of looking at the CGM data and evaluate how an intact pancreas can handle the different diets, and compare this with the controller used on the diabetic rats. However, in the case of the healthy rats, the controller is presented with glucose values from animals with their glucose regulatory system intact. It is then obvious that the OpenAPS suggest continuous low rate infusion of insulin, because the glucose levels do not really change much. With this in mind, how would you argue that you have evaluated the insulin levels of the healthy rats?

Responses: We agree with reviewer that we had not evaluated the insulin levels of the healthy rats. Performing these experiments, however, provided us with intriguing insights how well blood glucose is controlled in healthy rats regardless of food type and the stark differences in the patterns of BG and insulin infusion rate between healthy and diabetic rats. In the Discussion section, we added a paragraph discussing these points.

We have revised the manuscript incorporating our responses on p17, lines 450-457.

3. The results and the subsequent discussions are presented as one text. I am not convinced that this does the manuscript justice, and personally, I prefer them written separately. However, that is not for me to decide but if you choose to keep this way of presenting your findings, an idea could be to divide the section into separate subsections. This will help the reader to find the different results more quickly. Also make sure that the information is written in the appropriate sections, methods in the method sections and results in the result section.

Responses: Excellent suggestions! We divided Results and Discussion section into two separate sections. We added separate subsections in the Results to enhance readability. We also revised the manuscript to report the information in the appropriate sections.

We have revised the manuscript incorporating our responses throughout the manuscript.

4. The abstract is detailed and describes the major points of the manuscript well. The motivation for the study could perhaps have been more clearly stated. I believe that the aim is to describe the animal model for other researchers to use as a guide when planning their experiments, and not an evaluation of efficacy of the OpenAPS, per se, but a more clearly defined aim would benefit the abstract.

Responses: The abstract has been revised to include a clear aim of the study.

We have revised the manuscript incorporating our responses on p2, lines 26-27.

5. In the abstract, you suggest this animal model and technical set-up as a way to study glucose and insulin metabolisms, and different mathematical and engineering models of blood glucose prediction or new algorithms for artificial pancreas systems. However, in your conclusions, you explain that you are using this animal model to study the effect of sodium-glucose cotransporter-2 (SGLT2) inhibitors to treat diabetes type 2. Since this seems to be an important use of the animal model, you should include this kind of use in the abstract as well, as it is a good “selling-point” for the animal model.

Responses: We revised the abstract including various potential uses of the rat APS.

We have revised the manuscript incorporating our responses on p2, lines 48-49.

6. If I am correct in assuming that you want to describe this animal model for others to use in their research, there is a lot of important information missing both in the method section and in the result section. The crisis of reproducibility is a great challenge to all research, but perhaps particularly in animal research. I strongly recommend you to read and use the ARRIVE guidelines, which is a well-established set of guidelines to help with reporting data from animal research. More and more journals require authors to document that their reporting is in accordance with these guidelines. Below I have outlined some points in your manuscript that need addressing, but if you follow the guidelines you will be able to find more yourself, I’m sure. To start with, it is important to write your planned experiments in the method section, and describe the actual outcome in the result section. The results from this kind of study include much more than just blood glucose values and the amount of insulin used. The results include how the practical aspects of the experiments went, which is often overlooked in publications of animal studies.

Responses: We completed and submitted the ARRIVE Guidelines checklist and included descriptions of the practical aspects of the experiments as best as we could.

We have revised the manuscript incorporating our responses throughout the manuscript.

a. How many animals did you include in your planned experiments? You should describe this in the method section, both the total amount, the streptozotocin treated and the healthy animals, and how many animals you planned to use for the different experiments. In the result sections, you should start by describing how many rats you ended up using data from. Were any rats excluded? If so, for what reason? It is often a good idea to also include the number of animals in the tables.

Responses: A total of 30 (18 diabetic and 10 healthy, 2 euthanized) rats were used for the study. Specific number of rats used in each study is described in the Results section and the figure legends

We have revised the manuscript incorporating our responses on p5, lines 100-103 in the Methods section and figure legends of Figs 2, 3, 4, 5, 6)

b. How was the animal welfare evaluated? Did you use a score sheet? How did you define humane endpoints? This should be described in the method section. In the result section you should describe how the experiments actually went with regards to the animals and their welfare. Did they all tolerate the use of the restrainer, or did they show signs of stress? Did any incidents occur? Any infections?

Responses: Detailed descriptions of humane treatment of animals at all steps have been added in the Methods and the Results sections.

We have revised the manuscript incorporating our responses on p5, lines 104-113 in the Methods section and on p9-10, lines 218-226 in the Results section.

c. Were some animals used more than once? This is somewhat mentioned in the results section, but not clearly, and should first be stated in the method sections.

Responses: Yes, the same rat was used 2-3 times in order to minimize inter-variability and the number of animals used for the study for the majority of the experiments.

We have revised the manuscript incorporating our responses on p5, lines 101-103 in the Methods section

d. How long did you plan for each experiments to last? You mention that the rats were not placed in the restrainer for more not more than 3 days, but was that in total or per experiment? Did you have to cut some experiments short? If so, why? Your plans should be stated in the method section, and the actual result should be described in the result section.

Responses: For each experiment, a rat is placed in the restrainer for the duration of 2-3 days. Some experiments were cut short not due to concerns of animal welfare but technical issues such as sensor error, wifi connection, etc. Confinement in the restrainer 2-3 days causes mild stress for a rat shown by porphyrin rings around eyes despite acclimation process. In order to minimize the stress of a rat, an experiment was performed every two weeks for a rat. This is one of the limitations of the animal model and stated in the Conclusions section.

We have revised the manuscript incorporating our responses on p6, lines 133-136 in the Methods section and on p9-10, lines 218-226 in the Results section

e. I do not understand how the rat could be able move freely laterally in the restrainer, so a more detailed description of this would be nice. I have only experience with a restrainer made of more or less solid plastic and not metal bars, as I believe your restrainer consists of after a quick search on the internet. In my experience, the restrainer could not prevent the rats from rotating, but with a small modification of the tube we were able to prevent most of them from turning back-to front. How the rats were fixed during the experiments must be described in more detail in your manuscript, and a photo would be very beneficial.

Responses: A new figure (Fig 1) showing the overview of a rat APS system including the restrainer with a rat is added to the revised manuscript. Detailed descriptions of a rat’s mobility inside the restrainer were added in the Results section.

We have revised the manuscript by adding a new figure (Fig 1) and its descriptions (p9, lines 206-217) in the Results section.

f. Did you define criteria as to when a rat was considered diabetic? You write in line 107; “Rats developed full-blown diabetes in 1-3 days with BG greater than 500 mg/dl.” (This is actually a result and should be moved to the result section.) The predefined threshold should be stated in the method section and the actual result should be stated in the results section. Were some rats excluded because they didn’t become diabetic, or did they all become diabetic? Did any rats show any side effects of the streptozotocin treatment?

Responses: A rat with BG greater than 300 mg/dl was considered as diabetic. All rats injected with 60 mg/kg became diabetic in 1-3 days consistently. Two out of 30 rats were euthanized using vaporized isoflurane followed by injection of pentobarbital because the rats showed severe distress after streptozotocin injection. As an afterthought, severe hypoglycemia was suspected as the cause. After this incident, 10% sucrose water was given to rats for 1-2 days following streptozotocin injection until BGL > 300 mg/dl was observed. With this treatment, no more rats were lost.

We have revised the manuscript incorporating our responses on p6, lines 129-130 and on p5, lines 108-113 in the Methods section.

g. When was the different blood samples harvested (HbA1c, TAG, total-cholesterol, and HDL levels)?

Responses: After the studies that compared healthy vs diabetic rats, rats were fasted for 12 h and blood samples were harvested.

We have revised the manuscript incorporating our responses on p7, lines 167 in the Methods section.

h. How was the rats euthanized?

Responses: Rats were first anesthetized using vaporized isoflurane (5% volume-volume) mixed with oxygen, followed by injection of 200 mg/kg pentobarbital.

We have revised the manuscript incorporating our responses on p5, lines 104-107.

i. The comments and questions above are some points that you need to address, but the ARRIVE guideline will help you report your study and results more clearly. I also recommend you to use the PREPARE guidelines next time you are planning animal experiments. Together these guidelines help us improve our research, and I have great experience in using both of them.

Responses: Excellent suggestion! Will do.

7. What is “controlled conditions” mentioned in line 91?

Responses: Controlled conditions are temperature 68-73^o^F and 12 h light-dark cycle.

We have revised the manuscript incorporating our responses on p4, line 99.

8. What was the concentration of the isoflurane used for inducing anaesthesia (line 106 and 111)?

Responses: The concentration of isoflurane was 5% volume-volume.

We have revised the manuscript incorporating our responses on p5, line 104-105.

9. I am glad to read that you trained the rats so they were familiarized with the restrainer. How and for how long the animals were trained? And how long was the acclimation period before the start of the experiments?

Responses: Each rat was allowed to acclimate to stay in the restrainer gradually (6h, 12h, overnight) taking a few days prior to experiment.

We have revised the manuscript incorporating our responses on p6, lines 133-134.

10. When did you calibrate the CGM? Where and how did you collect the blood for calibrations? From the tail as you did for the analysis of TAG, total-cholesterol, and HDL levels? Did you cannulate the tail vein? How you collected blood samples should be described in the method section (and that goes for all blood samples). Did you calibrate the sensors at planned intervals? Was it necessary to calibrate them outside the planned schedule?

Responses: An Enlite sensor requires calibration 2h after insertion, followed by 6h initially, then every 12h. The sensor was calibrated a few times throughout the day following the manufacturer’s instructions. For CGM sensor calibration, a lancing device was used to draw blood (1-2 μl) from the tail. For lipid panel and HbA1C determination, rats were anesthetized using 5% isoflurane, a tail tip (1 mm) was cut, and blood (300-500 ul) was collected into 15 mg EDTA containing microfuge tubes.

We have revised the manuscript incorporating our responses on p9, lines 208-211 in the Results section and on p5, line 104-105 in the Methods section.

11. You state that you use the OpenAPS, but provide no further description. As the OpenAPS is a community based AP system, it constantly changes, as you also mention in the manuscript. You need to state which protocol you used, the date would perhaps be best. You should also state if you did or did not do any alterations or modification of the code/system.

Responses: OpenAPS algorithms are constantly changing as reviewer states. For this study we used oref0 0.6.1 and Nightscout (0.9.2). We had not modified OpenAPS algorithm (oref0 0.6.1). However, we modified Nightscout reporting view to analyze data on a specific time range.

We have revised the manuscript incorporating our responses on p9, lines 211-214 in the Results section.

12. In line 81, towards the end of the introduction you write; “An APS will provide a testing platform that is more dynamic and close to natural human physiology”. I believe that you meant to say that the AP systems you describe on live rats resembles human physiology more closely than in silico studies. The UVA/Padova simulator is widely used for in silico studies. It is approved by the FDA as a substitute for animal experiments on DM1 research, and it is based on human data from actual people with diabetes. I would appreciate a discussion of this statement in the manuscript, if and why you would trust the described animal model over, for instance, the UVA/Padova simulator.

Responses: In silico models such as the UVA/Padova simulator are extremely useful. However, there are innate limitations of in silico models because they often rely on the preset parameters of the virtual patients and make many assumptions. A rat APS test platform does not require making assumptions for modeling and simulation because it provides the input data on the fly.

We have revised the manuscript incorporating our responses on p4, lines 87-89 in the Introduction section.

13. With which statistical software did you analyse the data? And you should move the description of how you calculated AUC from “Intraperitoneal injection of dextrose” to “Statistical analysis”.

Responses: We used GraphPad Prism 6 statistics software and Microsoft Excel program. The description of how we calculated AUC was moved to the Statistical analysis section.

We have revised the manuscript incorporating our responses on p9, lines 199-200 in the Methods section.

14. In line 204 you write: “These studies suggest that stock concentration of insulin can potentially make a difference in controlling BGL in patients who use APS to treat type 1 diabetes.” Does the OpenAPS take into account which concentration of insulin is used, and make the calculations accordingly? If it does, do you have any ideas why different concentrations would influence the AP ability to regulate glucose?

Responses: Rapid-acting insulin formulations such as Humalog or Novolog are most commonly used for open or closed-loop OpenAPS. A sophisticated user may manually set the onset, peak, and duration of insulin action. Insulin stock concentration made a significant difference for closed-loop OpenAPS experiments in diabetic rats in terms of controlling BG. Its effect on humans is not known. In order not to make an overstatement, we removed the sentence.

We have revised the manuscript incorporating our responses by removing the sentence in the section where we discussed determination of appropriate dilution of insulin for a rat APS in the Discussion section.

15. In line 255, you describe how you injected dextrose solution in the IP space. Some of this text is actually a description of the method, and should be written in the appropriate part of the manuscript. You explain that this was performed in order to simulate unannounced meals. Does that mean that you informed the controller every time the rats ate by themselves? This is not described in the manuscript. How did you decide on the amount of carbohydrate in the IP injections? Clearly, they are larger than what rats normally would be able to absorb, as the healthy rats are not able to handle the large glucose load. Would you say that the IP injections of dextrose represents normal conditions (line 281)? Or are they supposed to representations of glucose elevations in humans?

Responses: We agree with reviewer that claiming IP injection of 50% dextrose as unannounced meal is not an accurate description. Accordingly, we changed it to ‘supra-physiological concentration of glucose’ instead of ‘unannounced meal’. The initial thought was to disturb the glucose-insulin homeostasis significantly in rats by introducing high glucose into the system, mimicking a large meal in humans. The highest amount of dextrose (4 g/kg) is twice as much as the amount used for intraperitoneal glucose tolerance test. For rats, this amount seems to be supra-physiological concentration. Healthy rats were able to handle it but it took much longer than 2h.

We have revised the manuscript incorporating our responses on p2, lines 39-40 in the Abstract and p18, line 458 in the Discussion section.

16. What are the units of the values presented in line 270?

Responses: The unit is mg/dl$\cdot$ min.

We have revised the manuscript incorporating our responses on p18, line 466 in the Discussion section.

17. What are the strengths and limitations of your study? A critical, but fair, self-evaluation of a study should always be a part of any research article.

Responses: The strengths of the study are establishing a rat APS that may be used as a novel research model to study a variety of biomedical and engineering approaches to enhance understanding and treatment of diabetes (described in the Conclusion section). The limitations are technical difficulties to set it up initially and limited number and frequency of experiments due to the duration of each experiment for 2-3 days and concerns of animal welfare (APS experiment is done every two weeks for a rat).

We have revised the manuscript incorporating our responses on p19, lines 505-507 in the Conclusions section.

18. In line 374, you state that these experiments are minimal invasive and can provide general physiological state of rats. This set-up is perhaps not very invasive, but highly interfering. How do you think the fact that the rat is restrained for 2-3 days might affect the results?

Responses: A rat placed in the restrainer is able to stretch head outside of the restrainer to eat food and drink water and move forward and backward. A rat is also be able to sleep with head down on the bottom of the restrainer. Urination and bowel movement are normal. However, rats show a mild sign of stress (slight porphyrin ring around eyes) by being confined in a small space despite the acclimation process. The porphyrin rings disappear in a day after termination of an experiment. In order to minize stress of being confined in a small space, experiment has been performed every other week for a rat for 2-3 days at a time. The frequency and the duration of experiment do not seem to affect the health of a rat greatly based on the change of bodyweight, eating, drinking, and grooming behaviors. In terms of comparing healthy and diabetic rats, the same condition of confinement is applied to both.

We have revised the manuscript incorporating our responses on p9-10, lines 218-226 in the Results section.

19. It is nice to see how the screen of the OpenAPS system looks like and how it is presenting relevant data. However, for this manuscript it does not provide enough data. There is no y-axis for insulin infusions, and the y-axis for BG values are not complete with units. It is nice that you have made a separate figure to present the BG values after IP injections of dextrose. Perhaps the insulin infusions could be included in these figures?

Responses: Insulin infusion pattern is shown in the Nightscout images. We agree that x-axis and y-axis label of these images are not clear. We remade figures with clear labeling of x-axis and y-axis.

We have revised the manuscript incorporating our responses by remaking Figs 6 and 7.

20. It is a strength of your study that you have examined the pancreas and liver post mortem, and present both written results and photos in the manuscript.

Responses: Thank you. We agree.

21. There is a statement that there are supplementary files, however, these have not been available to me. I apologize if my comments and questions are already addressed in the supplementary files. Have you included supplementary files? And where will you publish the raw data?

Responses: We have not submitted supplementary files. We apologize for the confusion. We are considering only Plos One for the publication of this study.

22. The text in the y-axis of some of the figures should perhaps be adjusted, for esthetical reasons

Responses: We remade figures of multi-panel ones (Figs 6 and 7) to show y-axis more clearly and label x-axis.

We have revised the manuscript incorporating our responses by remaking Figs 6 and 7.

23. This is no big deal, but I believe PLOS One prefers the abstract to be without abbreviations if possible.

Response: We removed unnecessary abbreviations in the abstract except the ones repeated several times (i.e. BGL, APS).

We have revised the manuscript incorporating our responses by removing unnecessary abbreviations throughout the abstract.

24. In the short title, blood is written with a capitol letter but not insulin is not.

Response: Correction has been made.

We have revised the manuscript incorporating our responses on p1, line 6.

25. Is the name of your institution correctly written in line 90? Should “Pharmacy” be written with a capital P?

Response: Thank you for noticing the typo. It has been corrected.

We have revised the manuscript incorporating our responses on p4, line 98.

26. I am not familiar with the term; “full-blown diabetes” in scientific literature. Is it a commonly used term?

Response: Yes, it is a commonly used term, interchangeable with ‘overt diabetes’.
