# Supplementary material for: Study of blood glucose and insulin infusion rate in real-time in diabetic rats using an artificial pancreas system
Source: PLoS One. 2021 Jul 16;16(7):e0254718. doi: 10.1371/journal.pone.0254718 (PMC8284668; doi:10.1371/journal.pone.0254718)
Supplement: Attachment — Submitted filename: Response to Reviewers-Final.docx [file pone.0254718.s002.docx]

**Response to Reviewers (PONE-D-21-07395).**

We sincerely appreciate Reviewers’ excellent critiques and their valuable time in reviewing our manuscript thoroughly.

Below are our responses to the critiques written in red, and revisions incorporated in the manuscript in blue. The page and the line numbers correspond to the marked version of the revised manuscript.

**Reviewer 1**

1. Several experiments were conducted under non-physiological condition. For instance, in Fig. 6, blood glucose level was over 400 mg/dL, and the authors did not monitored the exact level.

Responses: For intraperitoneal glucose tolerance test, 2 g/kg of glucose is commonly used. In order to observe how healthy and diabetic rats handle much higher concentration of glucose, we injected up to 4 g/kg of 50% dextrose, which allowed us to see much more significant differences between healthy and diabetic rats as opposed to using lower concentrations of glucose (2 g/kg or 3 g/kg). The plateau of BGL at 400 mg/dl is due to the upper limit of BGL measurement of Enlite CGM sensor. The comparison of healthy and diabetic rats injected with 3 different concentrations of glucose using the same system provided us valuable insights albeit this limitation. We do agree with Reviewer 1 that the upper limit of 400 mg/dl of BGL measured by Enite sensor can be a limitation for a certain study. Therefore, we clarified this in the Conclusions section.

We have modified the Conclusions section by adding this limitation shown on p 19, lines 488-489.

2. The authors might not have conducted statistical analyses properly. In the response to us, the authors mentioned that they used paired t-test and Mann-Whitney U test. Why did the authors analyze with both parametric and non-parametric methods?

Responses: We performed Mann-Whitney tests for comparing diabetic vs. healthy fed with chow or sugar pellets with respect to Normal % and Hypoglycemia % because these dependent variables were not normally distributed. Other than these 4 sets, the variables passed the normality test and thus we performed t-tests.

We have revised the manuscript incorporating our responses on p9-10, lines 223-227 in the Methods section.

3. In Table 1, standard deviation (or standard error) was not shown.

Responses: In Table 1, standard error of mean (SEM) has been added and mentioned in the Table 1 legend.

We have revised the manuscript incorporating our responses on p13, lines 314-317.

**Reviewer 2**

1. I appreciate the division of the Results and Discussion section. However, the Result section now begins with; “Establishment of a rat APS system” which is more appropriate in the Method section.

Responses: The description and the photo image of a rat APS system have been moved to the Methods section.

We have revised the manuscript incorporating our responses on p6, lines 131-144.

2. Where was the pentobarbital injected?

Responses: Intraperitoneally

We have revised the manuscript adding this information on p5, line 103.

3. Including figure 1 is very valuable to the manuscript, so thank you for including it. However, I believe is a photo, not a schematic diagram of the rat APS system. In the explanatory text, you write that the rat can move laterally, but have deleted this from the main text based on my previous comment.

Responses: We changed it to ‘A photo image of a rat APS system’ as a figure title. In Fig 1, we changed ‘laterally’ to ‘forward and backward’, which helps communicate better.

We have revised the manuscript incorporating our responses on p 6, line 143 and revising the text box on Fig 1.

4. I am glad to see that you have added the fact that there is concern for the welfare of the animals as a point in your manuscript. In line 225, you write that no experiments were cut short due to “concerns of animal welfare”, and in line 110 and 220 you describe some of the changes/symptoms expressed by the rats. Still, I cannot find your method for evaluating animal welfare or how you defined humane endpoints. If this was not done, I would question if the study was conducted in accordance with legislations and regulations (at least it would not have been in my country). This information should be included in all publications concerning the use of animals, and it is especially vital for this kind of study, which I would argue would be classified as a study with severe impact on the animals.

Responses: We defined the humane endpoints including the inability to reach food or water for approximately 12 h, a 20 % decrease in optimal body weight, a body condition score less than 3 on a 9-point scale, and significant pain cannot be alleviated by analgesics. We have previously described in detail rats’ ability to drink, eat, sleep, normal bowel movements, and move albeit limited.

We have revised the manuscript incorporating our responses on p6-7, lines 149-152.

5. In line 391-392, you make a point of how this animal model provides “a versatile and dynamic in vivo testing platform close to real-life situation”. I agree that this model offers certain advantages for studying different aspects of diabetes and potential treatment options, I would, however, argue that the model does not provide “close to real-life situations”. Of course “close to” can be interpreted in many different ways. However, you are trying to mimic normal human situations. A rat fixed in a restrainer for three days differs greatly from a normal life even for a rat, and definitely from a normal human life situation. Concerning the severe impact on the welfare of the animals included in such a model, I encourage a more realistic presentation of the animal model. However, I appreciate that there would be different views on this topic, and that we might not agree.

Responses: Reviewer 2’s comments are well taken. We removed ‘close to real-life situations’ and added ‘testing platform that allows to study the impact of APS algorithm in controlling BGL of a rat in real-time’.

We have revised the manuscript incorporating our responses on p16, lines 421-422.
